# Why prudence is needed when interpreting articles reporting clinical trial results in mental health

**DOI:** 10.1186/s13063-017-1899-2

**Published:** 2017-03-28

**Authors:** Rafael Dal-Ré, Julio Bobes, Pim Cuijpers

**Affiliations:** 1 Clinical Research, BUC (Biosciences UAM+CSIC) Program, International Campus of Excellence, Universidad Autónoma de Madrid, Ciudad Universitaria de Cantoblanco, c/Einstein 3, 28049 Madrid, Spain; 2 Department of Psychiatry, School of Medicine, University of Oviedo, C/ San Francisco, 1, 33003 Oviedo, Asturias Spain; 3 CIBERSAM, Madrid, Spain; 4 Department of Clinical, Neuro and Developmental Psychology, Vrije Universiteit Amsterdam, De Boelelaan 1105, 1081 HV Amsterdam, The Netherlands; 5 EMGO Institute for Health and Care Research, Amsterdam, The Netherlands

**Keywords:** Clinical trials, Meta-analysis, Psychotherapy, Pharmacotherapy, Publication bias, Outcome reporting bias

## Abstract

**Background:**

Clinical trial results’ reliability is impacted by reporting bias. This is primarily manifested as publication bias and outcome reporting bias.

**Mental health trials’ specific features:**

Mental health trials are prone to two methodological deficiencies: (1) using small numbers of participants that facilitates false positive findings and exaggerated size effects, and (2) the obligatory use of psychometric scales that require subjective assessments. These two deficiencies contribute to the publication of unreliable results. Considerable reporting bias has been found in safety and efficacy findings in psychotherapy and pharmacotherapy trials. Reporting bias can be carried forward to meta-analyses, a key source for clinical practice guidelines. The final result is the frequent overestimation of treatment effects that could impact patients and clinician-informed decisions.

**Mechanisms to prevent outcome reporting bias:**

Prospective registration of trials and publication of results are the two major methods to reduce reporting bias. Prospective trial registration will allow checking whether they are published (so it will help to prevent publication bias) and, if published, whether those outcomes and analyses that were deemed as appropriate before trial commencement are actually published (hence helping to find out selective reporting of outcomes). Unfortunately, the rate of registered trials in mental health interventions is low and, frequently, of poor quality.

**Conclusion:**

Clinicians should be prudent when interpreting the results of published trials and some meta-analyses – such as those conducted by scientists working for the sponsor company or those that only include published trials. Prescribers, however, should be confident when prescribing drugs following the summary of product characteristics, since regulatory agencies have access to all clinical trial results.

## Background

It is well known that the reliability of published clinical trial results is far from optimal [1, 2]. Reporting bias is a structural problem present in both publicly funded and privately sponsored trials. It is primarily manifested as publication bias (many trials are not published at all, mainly because they did not yield positive results) and outcome reporting bias or selective outcome reporting (only outcomes or analyses yielding positive results are published). Both types of bias typically result in an overestimation of the benefits and an underestimation of the risks. This could easily lead to erroneous therapeutic decisions by clinicians and patients.

What it is not so well known is that the two disciplines dealing with mental health, psychology and psychiatry, are at the very top of the ranking of all natural and social sciences with regards to publication bias: 91.5% of all articles publish positive results [3]. This finding was already described more than 50 years ago when Sterling [4] showed that 97% of all published studies on psychology rejected the null hypothesis. More recently, an unusually high prevalence of psychology studies published with a *p* value of just below 0.05 has been observed: the number of studies with a *p* value of between 0.045 and 0.05 is much higher than that expected [5]. Furthermore, in 9% of psychology trials reported *p* values are inconsistent with the reported statistic [6].

## Clinical trials on mental health interventions and reporting bias

Clinical trials assessing mental health interventions are prone to two major methodological deficiencies that, ultimately, contribute to the publication of unreliable results. The first deficiency relates to the low number of trial participants: the low statistical power associated with small numbers of participants facilitates the finding of false positive results and exaggerated size effects [7, 8].

A search conducted on ClinicalTrials.gov [9] on 9 December 2015 on clinical trials on psychotherapy, showed that 909 trials were registered and that the median number of participants among 91 trials randomly chosen from those 909 was 120; however, the median number of patients on 43 pivotal trials on drugs for psychiatric conditions approved between 2005 and 2012 in the US was 432 [10], i.e., 3.6 times higher. In addition to that, a recent study found that more than 50% of 100 psychology trials could not be replicated; furthermore, the mean effect size among replicated studies was half of that described in the original articles [11]. Of note is that a low number of trial participants could also yield to false negative results that, if published, could mislead clinicians.

The second deficiency refers to the use of outcomes requiring a certain grade of interpretation, such as psychometric scales: since these require a subjective assessment, they are prone to remarkable variability depending on the implemented analytical option [7, 8]. In these cases, in a trial with a small sample size, the magnitude of the estimated effect could vary (the so-called ‘vibration effect’) and will depend on factors such as the principal endpoint (psychometric scale) chosen, the use of adjustments for certain confounders and the availability of alternative options in statistical approach [12].

Publication bias is common on mental health trials. Thus, in clinical trials on treatments of psychiatric conditions considerable publication bias has been found. In major depression a reduction of 25–29% of the effect size of psychotherapy was observed when adding the results of unpublished trials to those to published trials [13, 14]. Publication bias and selective outcome reporting have been also described for drug trials for major depression [15], anxiety disorders [16] and schizophrenia [17] in which sponsor companies decided to mainly publish positive trials, outcomes and analyses.

Safety information is rarely reported in both drug and psychological intervention trials. Outcome reporting bias of key safety results is common in trials on antidepressants and antipsychotics: when comparing the data included in the corresponding clinical trial registries, only 57%, 38%, and 47% of serious adverse events, cases of deaths or suicide were reported in articles, respectively [18]. In psychotherapy the picture is remarkably worse since harms of the interventions are rarely reported: possible or actual adverse reporting is 9 to 20 times more likely in pharmacotherapy trials than in psychotherapy trials [19]. All these biases have a considerable impact on the benefit and risks reported for mental health interventions.

## Network meta-analyses on mental health intervention trials and reporting bias

The relevance of reporting bias is much greater if these are carried forward to meta-analyses, a key source for clinical practice guidelines. In disorders having a number of commercially available drugs, but with few head-to-head comparative trials, it is common to conduct network meta-analysis. In these, investigators aim to rank all medicines after conducting direct and, mostly, indirect comparisons between available drugs. In a network meta-analysis with trials of antidepressants versus placebo, publication bias modified the ranking order of the first three drugs if unpublished trials were taking into account [20]. This was not the case in a network meta-analysis with antipsychotics, most likely due to the use of head-to-head comparative trials and the fact that publication bias in antipsychotic trials is less common than with antidepressants [21].

## How to prevent reporting bias

There are two major methods to reduce trial results’ reporting bias. One is to prospectively register the trial or systematic review or meta-analysis in a public registry before the start of the trial or review. Trials could be registered in a number of registries that accept both trials on medicines and psychotherapy such as, for instance, ClinicalTrials.gov [9] or ISRCTN [22], whereas systematic reviews could be registered on International Prospective Register of Systematic Reviews (PROSPERO) [23]. The other method is to publish all the results obtained. Both are described in the Consolidated Standards of Reporting Trials (CONSORT) Statement [24] for trials and the Preferred Reporting Items for Systematic Reviews and Meta-Analyses (PRISMA) guidelines [25] for meta-analyses. However, these guidelines for reporting outcomes are rarely required by psychiatry journals [26]. Prospective trial registration will allow checking whether they are published (so it will help to prevent publication bias) and, if published, whether those outcomes and analyses that were deemed as appropriate before trial commencement are actually published (hence helping to find out selective reporting of outcomes). These two requirements are mandatory for medicine trials in the European Union and the US; this, however, is not the case for nonregulated interventions, such as psychotherapy [27]. However, this has changed but only for psychotherapy US National Institutes of Health-funded trials: in January 2017 a new policy on trial registration and publication of results has come into force [28], so psychotherapy trials will have to be registered and their results published. From an ethical perspective, the Declaration of Helsinki has also asked for these two requirements since 2008 [29].

Unfortunately, the rate of registered trials is low – only 25% of psychiatry journals ask for preregistration of trials to have the results published [26] – and, in many cases, of poor quality. Thus, among the top five psychiatry journals, that also ask for preregistration of trials, only 33% of 181 trials published in 2009–2013 were registered before the onset of participant enrollment, whereas only 14% had, in addition, no outcome reporting bias [30]. In other analysis, among 170 trials on depression published between 2011 and 2013, only 33% and 20% of trials assessing drugs and cognitive behavior therapy were appropriately registered (i.e., before study start and reporting fully specified outcomes), respectively [31]. With regards to psychotherapy, a recent systematic review on 112 randomized clinical trials published between 2010 and 2014, showed that only 18% were prospectively registered and only 5% were free of outcome reporting bias [32].

## Reporting bias and clinical practice

Prescribers should interpret the results of clinical trials and meta-analyses with caution: publication in a prestigious journal does not prevent selective reporting of outcomes. It would seem reasonable to expect that journal editors should implement rigorous quality control mechanisms to prevent outcome reporting bias [33]. Because that is not implemented yet, clinicians should have a certain degree of skepticism with regards to all clinical trial results, irrespective of the types of intervention assessed. Clinicians cannot be expected to compare the information included in an article with that provided on the trial registry. On the other hand, although there are a number of methods to explore whether a meta-analysis presents any type of bias [34], they are not feasible for the vast majority of clinicians. Prescribers should be especially skeptical when reading the results of meta-analyses when (1) scientists of the sponsor company were involved [35], and (2) no unpublished trials are included, since this usually implies a variable impact on the direction and size of the therapeutic effect [36].

It should be highlighted, however, that since regulatory agencies have access to the results of all clinical trials with medicines, the clinician should be confident when filling a prescription following the authorized summary of product characteristics. The situation is very different with trials on off-label indications, where selective outcome reporting is common [37], and with trials conducted not to amend the approved indication, posology or target population of a drug, but to inform prescription habits (e.g., comparative effectiveness trials) that are not subject to regulatory agency in-depth review: articles of these two types of trial are subject only to the peer-review process which has no impact in rejecting for publication manuscripts with discrepancies in registries [38]. Clinicians should be even more skeptical when reading psychotherapy trials that, since they are not regulated, could easily present outcome reporting bias of both benefits and harms, hence hindering the benefit/risk assessment.

## Concluding remarks

The only way to ensure the absence (or minimization) of outcome reporting bias is by implementing better-quality control procedures during the editorial process, such as a thorough cross-checking between the manuscript and the protocol or registry [39]. Until this happens, clinicians should be prudent when interpreting the results of published trials and some systematic reviews/meta-analyses. This is based on the fairly frequent presence of outcome reporting bias that tends to overestimate treatment effects in mental health trials. As a nonregulated intervention, psychotherapy trials are especially prone to this fact. Pharmacotherapy is also subject to outcome reporting bias, but since regulatory agencies will have access to pivotal trials results, the summary of product characteristics is a fair description on how a drug can be correctly prescribed.
